# Novel *CHATogether* family-centered mental health care in the post-pandemic era: a pilot case and evaluation

**DOI:** 10.1186/s13034-024-00750-y

**Published:** 2024-05-21

**Authors:** Caylan J. Bookman, Julio C. Nunes, Nealie T. Ngo, Naomi Kunstler Twickler, Tammy S. Smith, Ruby Lekwauwa, Eunice Y. Yuen

**Affiliations:** 1 Department of Psychiatry, Yale University, New Haven, CT USA; 2 Cambridge Health Alliance, Harvard Medical School, Cambridge, MA USA; 3 Yale New Haven Hospital, New Haven, CT USA; 4 Yale Child Study Center, New Haven, CT USA; 5 Yale Department of Psychiatry, 300 George Street, 06511 New Haven, CT USA

**Keywords:** Children and adolescent mental health, Family mental health, Family-centered intervention, Intensive outpatient care, Post-COVID-19 pandemic

## Abstract

**Background:**

The COVID-19 pandemic impacted children, adolescents, and their families, with significant psychosocial consequences. The prevalence of anxiety, depression, and self-injurious behaviors increased in our youth, as well as the number of suicide attempts and hospitalizations related to suicidal ideation. Additionally, parents’ mental health saw increasing rates of depression, irritability, and alcohol use combined with worsening family function, child-parent connectedness, positive family expressiveness, and increases in family conflict. In light of these statistics, we created *CHATogether* (Compassionate Home, Action Together), a pilot family-centered intervention using multi-faceted psychotherapeutic approaches to improve familial communication and relational health between adolescents and their parents. This paper discusses the implementation of the *CHATogether* intervention at the Adolescent Intensive Outpatient Program (IOP), providing an example of the intervention through an in-depth pilot case, and evaluation of the program’s acceptability and feasibility.

**Methods:**

This paper describes a case in detail and evaluation from a total of 30 families that completed *CHATogether* in the initial pilot. Each family had 4–6 one-hour *CHATogether* sessions during their 6-week treatment course at the IOP. Before and after *CHATogether*, adolescents and their parents separately completed a questionnaire designed to explore their perceived family conflicts. After completion of the program, participants completed a brief quality improvement survey to assess their overall experience with *CHATogether*. In the reported case, the family completed Patient-Reported Outcomes Measurement Information System (PROMIS) depressive and anxiety symptoms scales, Conflict Behavior Questionnaires (CBQ), 9-item Concise Health Risk Tracking Self-Report (CHRT-SR9), and help-seeking attitude from adults during distress and suicide concerns.

**Results:**

The pilot case showed a trend of improvement in reported depressive and anxiety symptoms, child-parent conflicts, subfactors of suicide risk including pessimism, helplessness, and despair, help-seeking acceptability from parents for suicide concerns, and the establishment of individualized family relationship goals. Preliminary feedback from participating families demonstrated positive effects on intra-family communication and improvement in the overall family dynamic. Adolescents (*n* = 30/30) and their parents (*n* = 30/30) rated “strongly agree” or “agree” that their families had benefited from *CHATogether* and welcomed participation in future program development.

**Conclusion:**

This study presents *CHATogether* as a novel family-centered intervention to address post-pandemic family mental health stress, especially when a family system was disrupted and negatively affected the mental health of children and adolescents. The intervention facilitated positive child-parent communication on a variety of topics, through tools such as emotional expression and help-seeking behavior. The reported pilot case and evaluation suggested *CHATogether*’s acceptability and feasibility in a clinical context. We also provided quality improvement feedback to guide future studies in establishing the efficacy of *CHATogether* and other similar models of clinical family interventions.

## Introduction

Since the COVID-19 pandemic, both national and regional organizations have highlighted the significant increase in mental health challenges faced by children, adolescents, and their families. As a result, the American Academy of Child and Adolescent Psychiatry, in partnership with the American Academy of Pediatrics and the Children’s Hospital Association declared a national emergency in children’s mental health in 2021 [1].

This mental health crisis has been due in part to the stress and disruption within family systems, as well as the relative isolation caused by the various constraints of the pandemic. These factors resulted in a significant increase in the utilization of children and adolescent mental health services [2, 3]. The pandemic left a notable negative footprint on societal structures and dynamics, putting increased pressure and strain on individual families [4–6]. Many community support systems such as schools, places of worship, and recreational spaces were abruptly closed due to the pandemic, leaving families without vital community resources. The pandemic exacerbated existing inequalities for families with ethnic minority and low socioeconomic backgrounds [7]. Taken together, these factors disrupted parental reflective functioning and adaptive family communication [8]. The pandemic forcefully imposed a new norm – social isolation, boredom, elevated parental scrutiny, and the loss of independence – all of which negatively altered how a child or teen reacted to their parents [4–6].

Furthermore, there were notable changes in children’s and adolescents’ behaviors, such as an increase in difficulty concentrating, lower frustration tolerance, and a lower threshold for “general discomfort” [9, 10]. Parents stepped into multiple new roles at home, attempting to meet the increased needs of their children. Simultaneously, they worried about health and finances, leading to increased frustration and authoritarian parenting, decreased engagement with their children, and ability to reason and self-regulate [4, 11].

In 2019, we established *CHATogether* (**C**ompassionate **H**ome, **A**ction **T**ogether), a digital community-based mental health program tailored for children, adolescents, and their families. *CHATogether* included different creative modalities to address cross-cultural and cross-generational needs while also promoting improved mental health among adolescents and their families. The program consisted of six core arms that include: digital interactive theater, public mental health education, research, peer support and community outreach, collaboration, and mentorship. These arms aimed to provide a feasible and successful family wellness initiative in response to the COVID-19 pandemic [12]. Through this community effort, we have witnessed the dire clinical need to address familial mental health as a one-unit system during the post-pandemic era.

In 2022, we have adapted and implemented the clinical *CHATogether* program at the adolescent Intensive Outpatient Program (IOP), a subacute level of mental health care that supports both adolescent patients and their families. During the IOP intake assessment, adolescents and families were routinely asked about their perception of parenting practices. Our clinical impression suggested that most families perceived each other to have challenges in at least one or more domains of effective parenting (e.g., consistency, supervision/monitoring). Moreover, a significant discrepancy between the adolescent’s and parent’s perspectives regarding these domains implicated opportunities to bridge this unmet clinical gap and improve the overall adolescent-parent dyadic functioning. This pilot evaluation aimed to (1) report on the *CHATogether* family-centered intervention, (2) provide a pilot case with preliminary data to exemplify how the intervention works, and (3) evaluate the acceptability and feasibility from a total of 30 families who completed *CHATogether* in the adolescent IOP during the post-pandemic era.

## Methods

### Theoretical context of *CHATogether* family intervention

*CHATogether* is based upon a psychodynamic, bio-psycho-social model that focuses interventions on the family/social systems aspect of this dynamically interactive tripartite model. It incorporates psychotherapeutic elements from drama therapy, cognitive behavioral therapy (CBT), and psychologically focused therapies in ways that improve family relational health and communication, and adolescent mental health. The program facilitated and guided family members’ expression of unconscious conflicts, feelings, fears, and imaginings through engaging in theater skits and role-playing [13–15]. Drama therapy techniques allowed participants to project their internal feelings and conflicts onto skit characters [16–18]. As in psychodynamic play therapy, participants could experiment within the safety of displacement that theater provides [19, 20]. Previously intolerable fears as well as emotionally charged feelings would be identified, become available for collaborative reflection, and lead to eventual conflict resolution. Drawing upon aspects from psychodynamic, CBT, and drama therapy, the clinician helped families develop better adaptive, healthier patterns of communicating feelings and behaviors as illustrated by the skits [21, 22]. Additionally, the clinician made drama-to-life connections whereby family participants could apply what they learned to improve communication patterns amongst themselves.

*CHATogether* has emphasized a whole family approach [23, 24]. Thus, it could be a therapeutic process that addresses relational conflicts within the family system, the third component of the biological-psychological-social/family model, in addition to the biological and psychological treatments the adolescents typically receive in IOP. Traditional family therapies could have low adherence due to factors such as families’ resistance to engage in treatment that require multiple sessions over many months for meaningful change to be accomplished [25, 26]. Compared to the inpatient services or outpatient clinics, the IOP has been the ideal setting for implementing high-impact, brief family interventions given the acuity and need of the population as well as the time frame and highly structured nature of IOP treatment [27].

**Table 1:** Participating families’ demographics

	No. out of 30	%
Age of index child (range)	15.5 (12–17)	
Sessions completed (range)	5.4 (4–6)	
Gender		
Male	12	40.0
Female	14	46.7
Non-confirming	4	13.3
Primary psychiatric diagnosis		
MDD	10	33.3
GAD	8	26.7
PTSD	3	10
OCD	4	13.3
Adjustment disorder with mixed anxiety and depressed mood	3	10
ADHD	2	6.7
Race		
White	12	40
African American	5	16.7
Hispanic/Latino	4	13.3
Asian American	5	16.7
More than one race	4	13.3
Referral sources		
Psychiatric inpatient	14	46.7
Emergency room	10	33.3
Outpatient service	6	20
Living arrangements		
Both parents	14	46.7
One parent	8	26.7
Alternating residences	5	16.7
Extended family	3	10
Program participation		
Two parents	14	46.7
Mother only	10	33.3
Father only	4	13.3
Other caregivers	2	6.7
Parental marital status		
Married	12	40
Divorced or separated	17	56.7
Widowed	1	3.3
Program delivery format		
In-person	16	53.3
Virtual	8	26.7
Hybrid	6	20

*MDD* major depressive disorder, *GAD* generalized anxiety disorder, *PTSD* post-traumatic stress disorder, *OCD* obsessive-compulsive disorder, *ADHD* attention-deficit/ hyperactivity disorder

### Participants recruitment and selection criteria

A total of 30 families with adolescents aged 12–17 years old were included in this pilot assessment of the *CHATogether* program (Table 1). The 30 participating families included teens (*n* = 30), who were accompanied by both parents (*n* = 14), mother only (*n* = 10), father only (*n* = 4), or other caregivers (*n* = 2). All adolescents were existing patients enrolled in Yale New Haven Hospital (YNHH) Adolescent Outpatient Behavioral Services from October 2022 to December 2023. Patients attended the conventional 6-week general mental health intensive outpatient program (IOP) track, which included 3 hours per day, 4 days per week of after-school group therapy, weekly medication management, and family case management for discharge planning. The goals of the general IOP emphasized the stabilization of psychiatric symptoms and safety through treatments such as medication management and learning coping strategies from group therapy. The IOP treatment patients were referred from the YNHH inpatient adolescent units, emergency department, Yale Child Study Center, and local outpatient clinics in Connecticut.

Regarding inclusion and exclusion criteria, this study included patients who have significant family relational conflicts that may hinder the conventional IOP treatment. This IOP does not typically accept patients with high acuity psychiatric conditions, including those with significant active psychosis, substance intoxication/withdrawal, active eating disorder, delirium, or developmental delay with significant cognitive impairments as these conditions can impede the ability of adolescents to fully engage in treatments. In the study, *CHATogether* participating adolescents and their parents/caregivers received additional treatments through at least 4–6 one-hour family sessions during the 6-week IOP. Parental consent and adolescent assent were discussed at least one week prior to the intervention.

### *CHATogether* family session protocol

The program was delivered in-person, virtually, or using a hybrid format, depending on the parents/caregivers’ availability. A total of 3 clinicians delivered the intervention. Pre- and post-session questionnaires were individually completed by adolescents and their parents/caregivers in the first and last session of the *CHATogether* program.

Partly adopted from the Family Relational Assessment Protocol (FRAP) [28], the questionnaires included family relationship goals, major conflicts in the child-parent dyads, major conflicts between the parents, family losses, and major areas to change. The design encouraged the families to revisit the questions and troubleshoot together with the newfound strategies and communication (Table 2). The first *CHATogether* family session consisted of guiding the family to view a three-part theater skit in the following order: (1) problematic scenario; (2) pause and moderation; and (3) alternative scenario (https://www.youtube.com/watch?v=6Rx7D4x1nLY).

### Problematic scenario: “A Christmas Carol epiphany in child-parent relation”

Mentalization is an essential capacity to envision the state of mind in self or others, which involves understanding and anticipating each other perspectives through a lens of curiosity and without judgment [8, 29, 30]. According to this theory, mentalization not only allows parents to understand their child’s behaviors and underlying feelings, but also helps parents respond to the child’s emotional needs sensitively, and ultimately improve the child’s affect regulation [31, 32]. To introduce the concept of mentalization, the family watched the skit video, “*Parents Got All the Solutions*”. Prior to viewing the skit, parents or caregivers were prompted to mentalize the teens’ perspective in the skit, whereas adolescents were asked to mentalize the parents’ challenges in the skit.

A high school teen is trying to communicate to his father about his feelings of depression and thoughts of suicide. Being a single parent, the father in the skit has worked all day and is taking care of three children. The father is terrified because he has found several empty pill bottles in his teenager’s room and is now persistently offering “solutions” in an attempt to “fix” his son’s mental health.

### Pause and moderation: learn, share, and reflect

The clinician guided the family to identify unhelpful examples of communication in the problematic scenario. In the initial half of the moderation, therapeutic interruption and facilitation were employed by the clinician to help the family focus on discussing the skit instead of directly jumping into their family conflicts. First, verbal and nonverbal communication including language, word choice, tone, body gestures, and any expressive emotion that may impede a caring conversation were discussed. Second, the clinician pointed out parental invalidation in the video and stressed the importance of validating a teenager’s emotions before offering solutions or guidance. Third, the clinician introduced the concept of “listening to understand” rather than reacting to a conflict. Additional points for discussion included the acting teenagers’ attitude in the skit, for example, their tone of voice, frustration, lack of patience, and how these can negatively impact the parents’ understanding of the situation. Lastly, the clinician guided the family to relate their own experiences to the themes illustrated in the skit, allowing the family to draw parallels between the problematic communication styles in the video to ones possibly in their own family.

### Alternative scenario: establish commitment, communication exercises, and follow up

The alternative scenario of the skit “*Parents Got All the Solutions*” displayed the situation with more effective communication strategies. The family was prompted to compare and contrast the two scenarios. The clinician guided the family to reflect upon how they can practice healthy communication in their home life. Given its emotional intensity, the first *CHATogether* family session fostered the best opportunity for parents and their children to emotionally reconnect and establish a mutual commitment to positive change within the family. The session ended with facilitated cohesion where the clinician encouraged each family member to share their love and gratitude to each other. The family session concluded with the homework (a two-minute communication exercise every day) to be completed by the next session: (1) adolescents and parents commit a time to talk each day without stress and distraction; (2) each side takes turns to talk about their day and openly share their feelings without interruption for 2 min; (3) the listener practices mentalization while listening attentively and validating the speaker’s experiences afterward.

In the subsequent sessions, the family reflected on their communication exercises and the concepts introduced in the first session, leading to a shared learning experience. Each session also included psychoeducation on child-parent communication skills, signs and symptoms of mental health conditions illustrated from the initial skit, as well as a review of homework on skills/concepts discussed from the previous sessions. Moreover, the clinician emphasized any emerging relational conflicts in the past week and guided the family to practice their newfound communication and coping skills to reflect on or resolve the conflicts. If applicable, the clinician presented other skits involving different themes that followed a similar sequence to those described in the first session.

### Data collection

Institutional review board approval from the Yale Human Investigations Committee was obtained (Protocol #2000034837). Families consented to study participation prior to the beginning of the survey. The study was entirely voluntary, and the families were welcomed to receive *CHATogether* treatment regardless of their participation in the study. In the case vignette, additional written consent from the family was obtained, and the case was de-identified with modifications to protect the patient’s and family’s privacy.

To measure the program’s overall acceptability and feasibility in all participating families, a 6-item-survey 5-point Likert scale (“strongly disagree” to “strongly agree”) was delivered using the HIPAA-secured Yale Qualtrics system in the last *CHATogether* family session. Each question also included open-ended qualitative text boxes for participants to voluntarily elaborate on their answers (Table 3).

**Table 2:** Individualized family questionnaires

Questions	First *CHATogether* family session	Last *CHATogether* family session
What would be some family relationship goals you would like to accomplish? Rate severity 0–10 (10 being the farthest away from the goal)	Teen: “*Able to open up and share emotions with parents without them being reactive*” 9/10 Parents: “*Able to communicate without getting upset with each other.*” 9/10	Teen: “*My parents spend more time listening to me without jumping to conclusions.*” 1/10. Parents: “*She is able to come to me for good days, not so good days, and even crisis moments*”. 1/10
Identify major conflicts between you and your child, or you and your parents. Rate severity 0–10 (10 being the most problematic to the teen-parent relationship)	Teen: “*Phone being taken away due to concerns with online people*” 8/10 Parents: “*Phone taken away for safety reasons because she met boys through Snapchat and sent inappropriate pictures 10/10*”.	Teen: “*We problem solve and communicate how to keep me safe while letting me hang out with friends.*” 2/10 Parents: “*I see that she is more mature and better at judging what is right vs. wrong. Still work to do but better in communication*”. 3/10
Identify major conflicts between the parents. Rate severity 0–10 (10 being the most problematic to the teen-parent relationship)	Teen: “*Their parenting styles: mother is the discipline one; father is more supportive*” 8/10 Mother: “*Different parenting style and I am the one to do most of the work*” 7/10 Father: “*Our work schedule made it hard*” 7/10	Teen: “*They talk more calmly to each other without making me trapped in the middle*”. 1/10 Mother: “*More understanding about her stressors and we have better communication in parenting*”. 1/10 Father: “*We compromise and plan together. We gave each other feedback on parenting*”. 2/10
Identify a loss in your family. Rate severity 0–10 (10 being the most problematic to the teen-parent relationship)	Teen: “*Grandparents’ death from COVID affected the family a lot, but no one seems to talk about it*” 10/10 Parents: “*Lost from the COVID-19 isolation, loss of time of celebration, trip, etc, leading to conflicts within the family around her school*” 10/10	Teen: “*They share more about their own past and relationships with grandparents. I wish I had these conversations a long time ago*” 3/10 Parents: “*Now we make up the family together. I also have a better understanding about her struggles in school*” 1/10
Identify an area to change for your child, or for parents. Rate severity 0–10 (10 being the most problematic to the teen-parent relationship)	Teen: “*Give me space when I am not ready to talk without raising their voices*” 8/10 Parents: “*More motivated to her school and future plan” 10/10*	Teen: “*No more yelling. We have a way for me to come to them while they let me take space*” 3/10 Parents: “*I understand that her missing school because of her depression, but not making excuses. She now comes out to talk to me and the family instead of staying in her room all the time”. 3/10*

Parents: when both mother and father reported similar responses to the prompt

The study has been collecting validated psychometric measures for the study’s next phase since December 2023. Although not a significant sample size for statistical analysis in this paper, the existing families showed a similar trend to that described in Table 4. In the pilot case and Table 4, the teen filled out the following outcome measures in the first and the last *CHATogether* sessions. Measurements included Patient-Reported Outcomes Measurement Information System (PROMIS) depression and anxiety symptoms scales [33, 34], Conflict Behavior Questionnaires (CBQ) [35, 36], 9-item Concise Health Risk Tracking-Self-Report (CHRT-SR9) [37], and seeking adult help for distress and suicide concerns [38, 39]. The help-seeking attitude measurements included a three-part adapted version from Schmeelk-Cone et al. to detect (1) teens’ help-seeking acceptability from parents, (2) adult help for suicidal youth, and (3) reject codes of silence. The last item measured teens’ attitudes to resist the secrecy about their peers’ suicide concerns. A higher score indicated that the teen has a preference to reach out to an adult for help [38]. The case participant’s parents also completed the parent version of PROMIS and CBQ.

## Results

### Samantha’s case

Samantha was a 16-year-old girl who struggled with major depressive disorder (MDD), generalized anxiety disorder (GAD), and suicidal ideation. She never felt safe to verbalize her emotions or suicidal thoughts due to family conflicts and perceived judgment of mental illness. Her parents felt that Samantha’s suicidal thoughts were “wrong” and “unnecessary.” They also invalidated Samantha’s sadness from bullying she received at school which involved girl-to-girl relational aggression [40, 41]. Gradually, she secretly turned to online relationships where she was asked to share nude pictures with strangers. Several online friends proposed a suicide pact at a time when Samantha already felt emotionally distressed with nowhere to turn. Her parents never knew about these dynamics until Samantha disclosed it to her therapist, leading to her first psychiatric hospitalization.

After being discharged from the psychiatric hospital, Samantha continued to stabilize at the IOP. She either suppressed her emotions or quickly became dysregulated when her parents confronted her phone use. Her mind was occupied with dark thoughts that she did not deserve to live or that she needed to punish herself by self-harming. Similarly to before her hospitalization, none of these thoughts or actions were shared with her parents, as she believed this would result in being yelled at and criticized. She reported a complete distrust in her ability to keep herself safe. The family conflicts once led to a report to Child Protective Services concerning harsh disciplinary practices.

### The course of Samantha’s ***CHATogether ***treatment

In session 1, Samantha and her parents were shown the skit video, “*Parents Got All the Solutions*”, as described in the methods. Each member of Samantha’s family committed to make changes in alignment with the best interests of Samantha. Facilitating cohesion within the family, they were asked to share their love and gratitude at the end of the session. They then went home with simple two-minute daily exercises of active listening and mentalization without interruption. The clinician assigned the family similar exercises after each session as described in the method session.

In session 2, the clinician first reflected on the communication exercises and then began to address the pertinent areas of child-parent conflicts within the family as identified in Table 2. Samantha’s mother was concerned because Samantha spent too much time in her room on her phone with online strangers. Taking extreme safety measures, the mother took away Samantha’s phone, restricted all her social contacts, and ultimately did not allow her to leave home alone. Conversely, Samantha felt online friends mattered to her mental health, and was frustrated by her parents’ absolute control and lack of trust. This was an emotionally charged session that required the clinician to role-play in order to model the concepts of mentalization and validation. This demonstrated a more supportive and productive way to navigate conflicts. The clinician demonstrated possible conversing scripts for parents when Samantha expressed sadness from having no friends and being bullied at school. For example, the clinician instructed parents, “Let’s take Samantha’s perspectives by curiously envisioning her feelings, intentions, and behaviors without judgment or imposing parental assumptions (mentalization)”. “It must be really hard to face those mean bullies at school” (validation and empathy). “How can I support you to meet new friends while making sure that you are safe?” (collaborate to meet both Samantha’s and parents’ needs).

**Table 3:** Preliminary feedback from participating families (% agree or strongly agree on Likert scale)

Preliminary outcome measures (*n* = 30)	% Agree or strongly agree (Teen)	% Agree or strongly agree (Parent)	Representative quotes
1. Overall, I am satisfied with the *CHATogether* program we received	100	100	Teen: “Family benefited greatly from these sessions, and our communication and understanding of each other…and find the most effective ways to communicate.” Parent: “This was one of the most productive hours they have ever spend with professionals”
2. The services fit well with the existing IOP treatment and are convenient for us to participate in.	100	100	Teen: “My parents can do in-person and Zoom hybrid session to make possible in their working schedule.” Parent: “Glad to have virtual option available”.
3. As a result of *CHATogether* program, my family’s communication and functioning has improved.	100	100	Teen: “It was fun… I am more comfortable to share with my parents and did not feel being judged or criticized.” Parent: “Particularly enjoy the perspectives of helping child and parents to find a middle ground. “It changed my view of my child and as the result I am less irritable and frustrated in managing my child’s mental health condition.”
4. I will recommend *CHATogether* to others who may benefit from this program.	100	100	Teen: “Having someone to instruct family on how to best communicate, and having the doctor there to support me and moderate the sessions was very helpful. The personalized sessions (asking for goals from parents and the child and then working towards them) is also very helpful.” Parent: “This intervention upbrings issues that parents have overlooked with our children.”
	%Yes, No, or Maybe (Teen)	%Yes, No, or Maybe (Parent)	
5. Are you interested, or would you like to see your parent participate in *CHATogether* parent groups in the future?	73.3/3.3/23.3	80/0/20	Teen: “family sessions will be more helpful than parent groups.” Parent: “would love to learn more parenting skills and have other parents’ support.”
6. What are some other skit video topics would you like to include in the future?	Teen: “How to make time to communicate and understand each other perspectives” “Retore trust and respect in my mental health journey” “Learn coping skills to stay calm during our communication”		Parent: “Overall communication and taking mutual perspectives.” “How to communicate about substance use” “Understanding psychiatric condition and how we as parent can help.” “Learning ways to communicate psychiatric crisis.” “How to set and reinforce expectations with my child” “How to balance self-compassion as parents and emotional needs of my child”
7. Response rate (% of total participating families)	90.1	90.1	*A total of 33 families completed the program and 3 did not complete feedback study due to a lack of follow up. All data shown are based on 30 families.

**Table 4:** Samantha and her family’s clinical outcomes pre- vs. post-*CHATogether* intervention

Measures (*n* = 1 from the case)	Teen		Mother		Father	
	Pre	Post	Pre	Post	Pre	Post
PROMIS-depression (Raw/T-score)	40/61.1	14/41.1	27/59.2	11/32.1	37/69.7	11/32.1
PROMIS-anxiety (Raw/T-score)	39/63.8	13/38.6	29/64.8	20/54.8	33/68.9	10/34.4
CBQ (sum/max)	12/20	0/20	12/20	0/20	10/20	1/20
CHRT-SR9 (sum/max)	15/36	2/36	N/A	N/A	N/A	N/A
1. Pessimism	6/8	1/8				
2. Hopelessness	6/8	1/8				
3. Despair	3/8	0/8				
4. Suicidal Thought	0/12	0/12				
Help-seeking attitude from adults in distress and suicide concerns (sum/max)	28/36	36/36	N/A	N/A	N/A	N/A
1. Help-seeking acceptability from parents	6/12	12/12				
2. Adult help for suicidal youth	12/12	12/12				
3. Reject codes of silence	10/12	12/12				

*N/A* Not applicable in parent participants, *PROMIS T-Scores* < 55 = None to Slight, 55.0–59.9 = Mild, 60.0–69.9 = Moderate, ≥ 70 = Severe, *PROMIS* Patient-Reported Outcomes Measurement Information System, *CBQ* Conflict Behavior Questionnaires, *CHRT-SR9* Concise Health Risk Tracking Self-Report

In session 3, as the clinician reviewed the communication exercises, Samantha and her parents started to show mutual reflective capacity and acknowledged that everyone needed to empathize and compromise to become a functioning family. Samantha was willing to recognize her parents’ safety concerns were valid but wanted her parents to listen to her needs without getting angry. The parents recognized that Samantha needed to make friends and develop sound judgment before she would emerge into young adulthood. They started to understand Samantha’s suffering from mental illness and began to meet her emotional needs with less judgment and criticism. However, in areas of phone use, Samantha’s mother continued to emphasize rule-following responsibilities repeatedly, which once led to disagreement between the parents. The clinician then developed a contract with measurable items for the family to collaborate on phone use. For example, the parents practiced one-time questioning Samantha’s online friend on a given day, while Samantha proactively communicated with her parents about her friend such as his or her name, age, and location to ensure safety. By the end of the session, the family was able to articulate and elaborate on their gratitude to each other.

In sessions 4 and 5, Samantha’s mother gradually realized that she was fearful of letting Samantha make friends online due to the mother’s childhood trauma. To meet Samantha and her family’s needs, a video skit on “*Intergenerational Trauma: The Shark Music*” was shown. The clinician reflected on how intergenerational trauma can play a role in one’s perception of fear and safety, and how that may impact teen-parent communication and relational health. Samantha and her parents were surprised but also relieved by this deep conversation that could ever occur within a family, and how much they were able to view from each other’s perspectives. In the last session of *CHATogether*, Samantha and her mother conducted the post-session questions, reflected on their progress during the program, and discussed goals for their family. Towards the end of the treatment, Samantha developed a newfound interest in music, resulting in a shared interest between Samantha and her parents as they began participating in family music lessons together. Ultimately, Samantha returned to school and made new friends in the school’s music band.

As shown in the individualized family questionnaires (Table 2), Samantha and her parents’ relational health improved throughout 6 *CHATogether* family sessions. Samantha’s parents demonstrated a great improvement in their reflective functioning and began communicating with Samantha with curiosity instead of judgment. Parents were able to learn how to regulate their emotional stress while mentalizing Samantha’s emotional needs. Consistent with the trend of these narrative data in Table 2, Samantha’s and her parents’ scores improved across measures (Table 4). PROMIS T-scores decreased to the None to Slight range across informants by the post assessment. The family was able to communicate and develop a shared plan on phone use, engagement in healthy social relationships, and communication when a psychiatric crisis arises, as suggested in the reduction of CBQ reported by Samantha and her parents. Samantha’s CHRT-SR9 reduced, especially in the subfactors most closely associated with suicide risk [37], including pessimism, helplessness, and despair. There was no obvious change in Samantha’s attitude toward seeking help from a general adult during distress and suicide concerns (Item 2) but she had an increase in help-seeking acceptability from her parents (Item 1). Lastly, Samantha had no change in her attitude that youth struggling with suicide should not be left alone and should seek adult help even if a suicidal youth asked her to keep it secret (Item 3).

### ***CHATogether***’s preliminary feedback, acceptability, and feasibility

As shown in Table 3, preliminary feedback suggested that *CHATogether* is an acceptable and feasible intervention when implemented in family sessions with adolescents enrolled in the subacute IOP settings. Participating families (*n* = 30/30) strongly agreed or agreed that they were satisfied with the *CHATogether* treatment and that their communication and overall family functioning improved. Most of the participants felt that the program fit well with their existing IOP treatment, was convenient for their schedule, and would recommend it to others. There were 3 families who completed the program but did not follow up on the post-session questions after multiple contact attempts. There were 2 recruited families that did not complete the program as the patients were sent back to the inpatient unit due to the severity of their mental health. They did not return to IOP services after discharging from inpatients. Since we did not collect a complete set of data, these 5 families were excluded from the *n* = 30.

## Discussion

The COVID-19 pandemic has had serious mental health effects on children, parents, and the functioning of the family unit. The case of Samantha and preliminary feedback data from participating families indicated that *CHATogether* is an acceptable and feasible therapeutic intervention for adolescents who are experiencing significant family relational conflict and impaired communication in ways that contribute to symptoms and limit adaptive help-seeking behaviors. The pilot clinical impressions suggested that *CHATogether* can lead to more compassionate child-parent communication, an overall improvement in an individual patient’s functioning, and progress towards a family’s individual relationship goals. Our data also suggested that *CHATogether* is an acceptable and feasible pilot program to be implemented in the IOP systems using technology to allow in-person, virtual, and hybrid deliveries of care.

### A conceptualization of why ***CHATogether ***works

The *CHATogether* family-centered model has been a unique one. Adopted in part from the Brazilian playwright Augusto Boal’s “Theatre of the Oppressed” (TOp) from the 1970s [42, 43], it aimed to promote a non-hierarchical dialogue among participants to guide imagination-based changes and collective actions in a conflictual situation [44]. By projecting family conflicts onto characters in a theater skit, one possible explanation for the therapeutic effect of *CHATogether* is that it symbolically displaced participants’ unconscious internal world onto the tangible yet distant theater skits [13–15]. Such a modality, much like children’s play, may provide a safe means for displacing feelings, impulses, and imaginings too strong or potentially overwhelming to address directly [19, 20]. This approach could also provide participants with a window of access to unconscious and heavily defended emotions that become more consciously tolerable in the safety of displacement. Maladaptive defensive ways of coping with powerful emotions were identified and understood in ways that enhance perspective taking, effective communication, and possibilities for change. The case demonstrated how the clinician’s moderation facilitates a therapeutic process that leads to an ‘internal switch’ whereby parents experience the power of this “Aha! moment” of self-realization. Parents in the program identified this as one of the most compelling moments in the therapeutic process. Such pivotal points allowed parents to understand their children through different perspectives.

Once restored to a state of greater emotional stability, meaningful conversation between family members became possible. Another possible therapeutic benefit of *CHATogether* could be that the program coached parents in mindfulness practice [45, 46] which helped in reducing emotional reactivity and maladaptive, rigid defensiveness [31]. Ego strengths including self-observation were enhanced, identified, and supported. Some parents suggested that the skills they learned helped them remain attuned to their child’s emotional needs even while very frustrated. Most importantly, clinician moderation of the *CHATogether* program may enhance the process of mentalization [8, 29–32], facilitating mutual reflective functioning within the family. In the case of Samantha and her parents, they mentalized the experiences of the skit characters, mutually empathized with the characters’ challenges, and used this common ground and their newfound skills to improve their relationship. Such an approach could allow for the learning of multiple ways to better cope with their hyper- or hypo-aroused state of mind. Samantha and her parents were able to enhance their reflective functioning through cognitive curiosity and flexibility in their conversations with each other [31]. The program may improve reflective capacity not only between the teens and parents but also the relational health between caregivers, as Samantha’s parents reported improved communication when approaching parenting that resulted in less conflicts.

Each *CHATogether* session included a psycho-educational component illustrated by the contents illustrated in the skit. Integrating all three components of the bio-psycho-social model of mental health assessment and treatment, it was designed to address child-parent communication skills, signs and symptoms of psychological distress and disorder, as well as medication and its management. The clinician guided Samantha and her parents to identify the patterns of feelings, thoughts, and behaviors when comparing the problematic vs. alternative scenarios. Samantha’s family found that viewing, role-playing, and practicing homework exercises illustrated in the two skit videos, was especially useful in communicating better with Samantha [21, 22]. The measurement-based approach in tracking pre- vs. post-session rating scales also provided objective data to reflect on the family’s progress and highlight validations of everyone’s efforts in the treatment. Over time, *CHATogether* not only provided Samantha and her parents with coping skills and more adaptive defenses, but it also nurtured shared growth, love, and partnerships that were collaborative rather than adversarial in the face of difficult feelings. *CHATogether*’s therapeutic potential could be consistent with the family resilience framework which suggests the importance of shared belief systems in (1) meaning-making processes; (2) a positive, hopeful outlook, and active agency; and (3) transcendent values and spiritual moorings for inspiration, transformation, and positive growth [47–49]. This framework emphasized strengths and resiliencies within the broader context of family relationships and community resources in helping family members adjust to stress, and cope with loss. Throughout the *CHATogether* intervention, the family was asked to make a strong commitment to change, while aiming towards identified and shared family relationship goals. The program provided each family member with a means of processing their fears in ways other than unconscious ‘fight-flight’ patterns. It supported ways of managing more consciously experienced fears and grief, with less attacking of self or others. The *CHATogether* program may ultimately help to build family members’ capacities for compassion, hope, and resilience, thereby strengthening the family as a well-functioning unit.

### Family mental health: treating the whole family system

Cross-sectional research suggested that parents with self-reported higher stress levels had fewer positive parenting practices during the pandemic [4–6, 50]. Additionally, there could be the longer-term issue of family “scarring,” defined as prolonged problems in family relationships even after protective factors have been activated [51]. COVID-19 has led to an exacerbation of child-parent conflicts and household chaos [52]. Families have had an urgent need for interventions that can provide comprehensive support during and after times of crisis. *CHATogether* is a unique intervention in that it treats the whole family as the ‘patient’, and as one of three systems dynamically intertwined in the bio-psycho-social/family/community psychodynamic model of human development. Under this concept, the family should be the basis for health which catalyzes changes to improve the entire family and downstream individual functioning [23, 24]. At the systemic level, treating the family wholistically can also be helpful in light of workforce shortages to adequately meet the rising demands of children’s mental health services [53, 54].

The strength of the *CHATogether* program may be that it actively identified those factors that interfere with family functioning while therapeutically facilitating the love, cohesion, and deep devotion to one another that is the foundation of any family unit. This intervention could be especially vital for children’s and adolescents’ mental health when family function is compromised due to forces disrupting the security of jobs, food, finances, and health during the pandemic. With its focus on a deep respect for differing perspectives, *CHATogether* could calibrate the power dynamics within families to be more equitable and holistic.

### Limitations, challenges, and future directions

The current study was a pilot trial of an innovative family-centered treatment. We have illustrated a representative pilot case with supporting preliminary data and the acceptability and feasibility from a total of 30 families voluntarily enrolled in the *CHATogether* program implemented at the IOP level of care. Most patients were recruited within the state of Connecticut. There are several limitations and challenges that help guide future directions. The patient/family population in the current study may not be generalizable to larger populations. This preliminary study did not answer whether the improvement of child-parent communication was solely due to participation of *CHATogether* and/or in part from the conventional IOP treatment that does not include this family intervention component. Sustained impacts on the participating families beyond the 4–6 sessions of *CHATogether* were also unknown. Moreover, we did not collect more extensive qualitative data such as focus group and/or individual interviews, which would reveal more narrative information about the feedback.

Future studies should include randomized, mixed-method, and longitudinal investigations from larger and more diverse populations to compare *CHATogether* and conventional IOP vs. conventional IOP alone. Such a study could examine whether family-centered treatment may provide additional benefits in adolescents compared to those receiving biological- and psychological-based treatment. The inclusion criteria for the current study included patients who demonstrated family conflicts but were not specific to a diagnosis, symptoms, or level of functioning upon entry into IOP treatment. By collecting more data with validated psychometric measures, future studies for the *CHATogether* program as a family intervention model could examine whether participation in the program can contribute to the reduction of clinical symptoms and suicide in specific psychiatric conditions such as major depressive disorder and generalized anxiety disorder. The Inclusion of the established measures in reflective functioning within a family would be particularly valuable in the next stage of study [55]. In addition, reproducibility and scalability to deliver *CHATogether* could be a challenge. In this study, we have not measured the perception of the intervention’s acceptability and feasibility from the clinicians, but the intervention team meets monthly to collect verbal feedback as the program expands. We have produced tutorial videos and a standardized protocol to minimize inter-clinician discrepancy, and we also have plans to establish a nationwide webinar training series to elevate the program’s scalability. The program has been developing a digital library to provide access to *CHATogether* skit videos and training manuals to the trained clinicians.

While preparing this manuscript, we produced more skit videos based on varying child, adolescent, and family mental health topics. Future videos need to incorporate social determinants of health reflected in the diverse racial, ethnic, religious, and economic backgrounds of participating families. Translations with subtitles including Spanish and Chinese could be important. *CHATogether* skits were created based on diverse family scenarios with consultations from patients, their families, and the treatment team. The skit simulated yet may not be fully applicable to families’ real-life scenarios. Moreover, the “problematic scenario” from the intervention may re-expose unpleasant memories in patients and families, and thus anticipatory guidance and post-session emotional support would be essential. The intervention did not fully adapt to children and adolescents who are shy, have limited verbal communication, or feel more secure in the isolated digital existence. To this end, artificial intelligence with graphic illustrations, such as *Avatar Assistant*, *Digital Twins*, and *Virtual Self*, would be helpful venues for neurodivergent youth and their parents to better engage, identify, and express the emotions being depicted in the *CHATogether* skits [56–59]. Although it warrants thoughtful development in the future, AI-guided *CHATogether* chatbots could be a scalable solution for families with limited access to the intervention. Moreover, adapting *CHATogether* in a social media format like that of *TikTok* and *Instagram* could be developmentally palatable to the current generation of children and adolescents [60, 61].

## Conclusion

The long-term psychological turmoil that young people and their parents have endured in the past few years outlasted the official end of the COVID-19 pandemic. As young people navigated difficult emotions, parents also suffered from substantial mental exhaustion. Therefore, family-centered treatment could be critically needed during this era of post-pandemic children’s mental health. This pilot study suggested that *CHATogether* is an acceptable, and feasible model of therapeutic intervention for children and adolescents presenting with mental health disorders by: (1) including the family as a focus of intervention within the bio-psycho-social framework of assessment and treatment; (2) providing a safe space for family members to process painful and frightening emotions during a psychiatric crisis, (3) promoting perspective taking, reflection, and more mature defenses within and between family members under stress, (4) improving effective family communication and problem solving, especially during times of crisis, and (5) restoring family cohesion after disruption, through enhanced mutual understanding, compassion, and hope within the family unit. Although more extensive studies are warranted, the results of this pilot study demonstrated the promising potential for the *CHATogether* program to serve as an innovative family therapeutic intervention during the post-pandemic era and beyond.

## Data Availability

The data would be available from the corresponding authors upon reasonable request.
